# Enhancement of tibia bone and eggshell hardness through the supplementation of bio-calcium derived from fish bone mixed with chelated trace minerals and vitamin D3 in laying duck diet

**DOI:** 10.1016/j.vas.2021.100204

**Published:** 2021-09-08

**Authors:** Wirot Likittrakulwong, Sateanpong Moonsatan, Tossaporn Incharoen

**Affiliations:** a Animal Science Program, Faculty of Food and Agricultural Technology, Pibulsongkram Rajabhat University, Phitsanulok 65000, Thailand; b Division of Animal Science and Feed Technology, Department of Agricultural Sciences, Faculty of Agriculture Natural Resources and Environment, Naresuan University, Phitsanulok 65000, Thailand; c Center of Excellence for Agricultural and Livestock Innovation, Faculty of Agriculture Natural Resources and Environment, Naresuan University, Phitsanulok 65000, Thailand

**Keywords:** Bio-calcium, Chelated trace minerals, Eggshell hardness, Laying duck, Vitamin D3

## Abstract

Cracked eggs cause great economic losses in duck egg production. The use of eggshell-related vitamins and minerals is one of the most suitable approaches for solving this problem. Therefore, this study aimed to evaluate the effects of dietary bio-calcium derived from fish bone mixed with chelated trace minerals and vitamin D3 (BCD) on egg performance, egg quality and the hardness of the tibia bone and the eggshell in laying ducks. A total of eighty 30-week-old Khaki Campbell laying ducks were assigned to 4 groups. Experimental birds were provided a basal diet supplemented with 0.0 (T1), 0.5 (T2), 1.0 (T3), or 2.0 (T4) g/kg BCD. Our results indicated that a negative impact on egg performance was not observed (*P* > 0.05) in any dietary BCD groups. The different BCD levels had no significant effects on yolk color, yolk ratio, albumen ratio, eggshell ratio or eggshell thickness. Similarly, the calcium and phosphorus contents of the eggshell and tibia bone were not influenced (*P* > 0.05) by the dietary BCD. Tibia bone weight and length did not differ (*P* > 0.05) among the 4 treatments. However, tibia bone (*P* = 0.006) and eggshell hardness (*P* = 0.025) significantly increased and correlated with increasing BCD levels. The strongest tibia bone and eggshell were found in the 2.0 g/kg BCD group when compared to the control group (*P* < 0.01). Thus, the study concluded that the inclusion of 2.0 g/kg BCD mixture in laying duck diets can be a potential approach to improve tibia bone and eggshell hardness, without detrimental effect on egg performance.

## Highlights

• A combination of bio-calcium, chelated trace minerals, and vitamin D3 (BCD) might have synergistic effects that increase the amount of soluble calcium available for absorption and enhance the bone mineralization and eggshell calcification.
• The inclusion of BCD mixture in the laying duck diet can be a potential approach to improve bone hardness and eggshell strength.

## 1 Introduction

Eggs with cracked or broken shells before or during egg collection can be calculated as approximately 10 to 15% of all eggs produced (Stefanello, Santos, Murakami, Martins & Carneiro, 2014). Problems with eggshell quality cause great economic losses in industrial egg production. Thus, previous research has explored many strategies to improve the quality of eggshells, including genetic improvement, environmental control, and feed management. Adopting a nutritional strategy is one of the most feasible solutions for solving these problems. Quite a number of minerals and vitamins are essential for bone health and optimal eggshell characteristics, such as calcium, phosphorus, zinc, magnesium, and vitamin D. In particular, calcium is required primarily for several metabolic functions in poultry, for bone formation, eggshell production, and blood clotting (Nascimento et al., 2014; Rush, Angel, Banks, Thompson & Applegate, 2005). Including an optimal level and type of calcium in the diet is the most important nutritional factor, which directly affects the quality of the eggshell. Bouillon and Suda (2014) noted that egg-type poultry requires rapid calcium supply to the shell gland (uterus) for calcium deposition in order to produce hard-shelled eggs. A few studies have indicated that organic minerals are more bioavailable than inorganic minerals and could be added at a lower level (Liu, Ma, Zhao, Vazquez-anon & Stein, 2014; Mabe, Rapp, Bain & Nys, 2003).

In recent years, bio-calcium powder has been used in many nutritious food products, which is synthesized from the by-products of agricultural and food industries, such as the golden apple snail (*Pomacea canaliculate*), eggshell waste, and fish bones (Benjakul, Mad-Ali, Senphan & Sookchoo, 2017; Hassan, 2015; Laonapakul, Sutthi, Chaikool, Mutoh & Chindaprasirt, 2019). Jung & Kim (2007) recommended that bio-calcium powder derived from fish skeletons can be used as a highly soluble calcium source, which is likely to be introduced as a highly bioavailability calcium. It seems that the use of bio-minerals such fish bone calcium may be a novel option to increase the amount of calcium absorption via the intestinal mucosa. On the other hand, chelated trace minerals (manganese, copper, and zinc) are recently of more interest than the inorganic or organic minerals. The concentrations of trace minerals can be decreased when using a chelated form because of their high bioavailability. Vieira (2008) also noted that chelated minerals and vitamin C are strongly associated with mineralized bone matrix. Regardless, vitamin D is considered an essential vitamin for calcium absorption and bone mineralization, as well as eggshell calcification (Bouillon & Suda, 2014). It can contribute a vital role in bone metabolism (Rush et al., 2005), maintaining calcium and phosphorus homeostasis, skeletal health, and muscle development (Geng, Ma, Wang & Guo, 2018).

In Thailand, laying ducks were traditionally raised free-range in the open on harvested paddy fields. The golden apple snail was one of the main protein sources for free-range ducks, which also contains organic calcium carbonate (Tepsila & Suksri, 2018). Laonapakul et al. (2019) found that bio-calcium synthesized from golden apple snail shells had a higher CaCO_3_ and CaO content than bio-calcium from blood cockle shells. This suggests that their shells are a main source of bio-calcium for laying ducks rearing on paddy field. However, the government has gradually prohibited the free-range system in order to prevent the infection of avian influenza viruses carried by wild bird populations; consequently, all ducks are housed. For this reason, supplementing the diet with eggshell-related vitamins and minerals seems to be a suitable strategy for laying ducks raised in housing. While several studies have focused on the impact of including only a single vitamin or mineral, the use of a mixture of multiple vitamins and/or minerals may be more effective in improving eggshell quality due to synergistic effects. Therefore, the present study aimed to evaluate the effects of dietary bio-calcium derived from fish bone mixed with chelated trace minerals and vitamin D3 (BCD) on egg performance, egg quality, and the hardness of the tibia bone and the eggshell in laying ducks reared in closed houses.

## 2 Materials and methods

### 2.1 Animal trials and experimental diets

This research was conducted at the Poultry Nutrition Research Unit, Faculty of Agriculture Natural Resources and Environment, Naresuan University, Phitsanulok, Thailand. Twenty-week-old Khaki Campbell laying ducks were purchased from a commercial egg duck farm in Phitsanulok province, Thailand. All birds were placed in a climate-controlled room (using an evaporative cooling system) and fed a commercial diet (CP, 18%; ME, 2850 kcal kg^−1^) during the pre-experimental period. At 30 weeks old, a total of 80 Khaki Campbell laying ducks with similar laying uniformity were assigned to 4 experimental groups replicated 4 times with 5 birds per replication. Each pen was prepared so that 40% was slat floor and 60% was litter area (size 1.44 m^2^) similar, and each was installed with a water trough, feeder, and nesting box. The photoperiod was set to provide incandescent lighting of 10 lux under an automatic lighting program (similar 17L: 7D) during the feeding trial. Laying ducks in the control group (T1) were provided a corn-soybean meal basal diet without supplement (Table 1). The remaining 3 groups were given the same basal diet, supplemented with 0.5 (T2), 1.0 (T3), and 2.0 (T4) g/kg BCD. BCD supplement (Biocalcio™, Mezclas Biomix S.A.S., Colombia) was produced by mixing the bio-calcium, chelated trace minerals, sodium bicarbonate, ascorbic acid and Vitamin D3. Bio-calcium is a high bioavailability and produced from bone of bluefin tuna (source: South Pacific Ocean and Caribbean Sea). BCD mixture consisted of 197.00 g bio-calcium, 46.50 g sodium (as NaHCO_3_), 18.00 g manganese (as C_4_H_8_MnN_2_O_4_), 6.50 g zinc (as C_4_H_8_N_2_O_4_Zn), 2.25 g copper (as C_4_H_8_CuN_2_O_4_), 5.00 g ascorbic acid and 2,500,000 IU vitamin D3 (as calcitriol). These mixtures were supplemented with 0.5, 1.0, or 2.0 g per kg of the basal diet and administered to the broilers during the experimental period.

**Table 1:** Ingredients and calculated chemical composition of a basal diet (g/kg, as-fed basis unless stated otherwise).

**Items**	**g/kg, as-fed basis**
Ingredients	
Corn	524.0
Cassava meal	30.0
Vegetable oils	20.0
Soybean meal (460 g/kg CP)	212.0
Full fat soybean	105.0
Dicalcium phosphate (180 g/kg P)	22.0
Calcium carbonate	75.5
Salt	3.0
Vitamin-mineral premix^1^	3.0
L-Lysine	1.0
DL-Methionine	2.5
Mycotoxin adsorbents^2^	1.9
Pigment	0.1
Total	1000.0
Calculated chemical composition^3^	
Crude protein	175.0
Ether extract	61.2
Crude fiber	32.3
Calcium	35.1
Available phosphorus	4.2
Metabolizable energy (kcal/kg)	2850

^1^Vitamin-mineral premix provided per kilogram of diet: vitamin A (trans-retinyl acetate), 12,000 IU; vitamin D3 (cholecalciferol), 3000 IU; vitamin E (all-rac-tocopherol-acetate), 12 mg; vitamin K3 (bisulphate menadione complex), 3.6 mg; vitamin B1, 1.4 mg; vitamin B2, 5.4 mg; vitamin B6 4.2 mg; vitamin B12 (cyanocobalamin), 0.02 mg; nicotinic acid, 9 mg; pantothenic acid (D-calcium pantothenate), 9 mg; folic acid, 0.6 mg; biotin, 45 mg; choline chloride, 210 mg; selenium, 0.18 mg; cobalt, 0.3 mg; iodine, 1.08 mg; iron, 54 mg; zinc sulfate, 60 mg; manganese oxide, 96 mg; copper sulfate, 12 mg.

^2^Mycotoxin adsorbents is mixture of bentonites, illites, and kaolites, which chemically contain as following: 63.90% SiO_2_; 16.20% Al_2_O_3_; 1.95% CaO; 3.32% Fe_2_O_3_; 2.90% MgO; 3.90% Na_2_O; and 0.80% K_2_O. ^3^The nutrient values were calculated based on the analyzed nutrient values according toNRC (1994);

### 2.2 Assessment of egg performance and internal egg quality

All data were recorded during the 8-week experimental period to evaluate the egg performance and egg quality of laying ducks fed different levels of dietary BCD. Eggs were collected from each experimental pen 2 times per day (6:00 A.M. and 6:00 P.M.) and expressed as a number of eggs for one replication. Collected eggs were weighed and recorded daily, while remaining feed was measured weekly. Hen-day egg production, average daily feed intake, egg weight, egg mass, and feed conversion ratio were calculated on the basis of recorded data. During the feeding period, a total of 8 eggs from each group were collected weekly to evaluate the following parameters: eggshell thickness, eggshell strength, eggshell ratio, albumen ratio, yolk ratio, yolk color, and Haugh units. The eggs were broken onto a metal plate, and the height of the albumin was measured as the distance between the base and top of albumin. The albumin, egg yolk, and eggshell were weighed using a digital-precision scale (0.01 g), calculated and express as a ratio. Yolk color was evaluated visually by using the Roche Yolk Color Fan (Roche Lte., Basel, Switzerland). Eggshell thickness was measured at 3 locations (air cell, equator, and sharp end) using a digital micrometer (model MW200–01DBL, Moore & Wright Co., Ltd., UK). In order to determine the Haugh units, the eggs were hammered onto an aluminum plate, and the albumin height was measured as the distance between the surface of the plate to the top location of the inner thick albumen. The average of the obtained values was used in the formula suggested byIncharoen  & Yamauchi (2009): Haugh units = 100*log [H - 1.7W^0.37^ + 7.6], where: *H* = albumen height (mm) and *W* = egg weight (g).

### 2.3 Determination of tibia bone and eggshell hardness

A total of 8 eggs from each group were collected weekly to measure eggshell hardness. First, intact eggs were individually labeled and weighed using a digital balance. Whole eggs were vertically compressed on a Texture Analyzer (model QTS25, Brookfield Engineering Labs., Inc. Middleboro, MA 02,346 USA) at a constant speed of 10 mm min^−1^, according to the method of Incharoen, Wonnakom, & Khaskheli, 2018. To obtain the value for eggshell hardness, the maximal force of compression was recorded at the moment when the eggshell broke.

To measure the tibia bone, 4 birds from each group (1 bird/replicate) with the same uniformity were selected and euthanized at the end of the experiment to collect the tibia bones. The meat and connective tissues were cleanly removed from the tibiae, and then each sample was dried in an oven at 95 °C for 24 h. The weights and lengths of the right and left tibiae were measured in each bird. An average of these measurements was expressed as the mean weight or length of the tibia bone for all birds. The left tibiae were selected from each group and compressed on a universal testing machine (model 441, Instron, Ltd., England), according to the modified method of Nakhon et al. (2019). Maximal force (N) of constant compression was recorded after breaking the tibia bone to obtain a hardness value. Broken tibia bone samples were collected and kept in a humidity-controlled chamber until they were analyzed for calcium and phosphorus.

### 2.4 Analysis of calcium and phosphorus contents in eggshell and tibia bone

A total of 4 eggs from each treatment (1 egg/replicate) were collected and broken to obtain the eggshell samples before the end of the experimental period. Cleaned eggshell samples were dried in an oven at 95 °C for 24 h, ground into a fine powder, and kept in a dry box until analysis. The dried right tibia was finely ground using an electric grinder. To observe the surface morphology of both the ground eggshell and the tibia specimens, scanning electron microscopy (SEM; model JSM-5410LV, JEOL Ltd., Japan) was performed using high vacuum mode at a working distance of 20 mm and an accelerating voltage of 20 kV. In addition, the quantitative analysis of calcium and phosphorus in the eggshell and tibia bone was conducted by energy-dispersive X-ray spectroscopy (EDS; model Link ISIS 300, Oxford Instruments Ltd., UK), combining SEM with the backscattered electron.

### 2.5 Statistical analysis

Egg performance, egg quality, the hardness of the tibia bone and eggshell, and the calcium and phosphorus contents were analyzed by one-way analysis of variance (one-way ANOVA) using the Statistical Package for the Social Sciences (SPSS), version 17.0 (SPSS Inc., Chicago, USA). All results were presented as means of groups and the pooled standard error of the mean (SEM). Differences among the treatments were performed using Duncan's multiple range test. Probabilities (*P* < 0.05) were considered significant.

## 3 Results and discussion

The obtained results indicate that the average daily feed intake tended to be higher with increasing dietary BCD levels and was significantly increased (*P* < 0.05) in the 2.0 g/kg BCD group (Table 2). However, the addition of several levels of BCD in the laying ducks’ diet had no significant differences in regard to hen-day egg production, average egg weight, egg mass, and feed conversion ratio (FCR). In general, there is scant scientific information available to contribute to an understanding of the effect of dietary bio-calcium derived from fish bone mixed with chelated trace minerals and vitamin D3 on the productivity and eggshell quality of laying ducks. Our results indicated that a negative impact on egg performance was not observed (*P* > 0.05) in the dietary BCD groups. Previously, the elements of the mixture in poultry feeds has been reported singly, but not in combination. For example, a study of the effects of different calcium sources, from ground limestone or oyster shell, was investigated by Wang, Chen, Zhang, Ruan & Lin (2014)), who observed no significant differences in egg production, egg yield, and feed conversion in Longyan ducks over the whole 12-week period of the experiment. A recent study of the application of nano calcium carbonate showed that substituting calcium carbonate with nano calcium at levels of 0.126–2.015% had no detrimental impact on egg performance or egg quality in laying hens (Ganjigohari, Ziaei, Ramzani Ghara, & Tasharrofi, 2018). In addition, Gongruttananun (2011) reported that ground eggshell can be used as an alternative source of calcium without detrimental impacts on egg productivity, plasma Ca balance, bone mineralization, and gonadal performance in laying hens. Dietary supplementation of chelated trace minerals (Cu, Zn, and Mn) did not have a negative effect on growth performance or tissue mineral storage in broilers, thereby potentially reducing the excretion of minerals into the environment (Zhao et al., 2010). Carvalho, Rosa, Litz, Fagundes & Fernandes (2015) also reported that a mixture of the minerals copper, manganese, and zinc in the organic form could be used in layer diets as a replacement for inorganic sources, which had the potential to reduce trace mineral excretion without negative effects on egg production or eggshell quality. According to Adhikari, White, House & Kim (2020), who studied the feeding of different forms of vitamin D in laying diets, egg production and egg weight were not significantly different among the groups that received vitamin D2, vitamin D3, or 25-hydroxyvitamin D3. Dietary vitamin C exhibited the greatest laying rate in hens, even when reared under heat stress conditions (Attia et al., 2016).

**Table 2:** Egg performance and egg quality of laying ducks fed dietary bio-calcium derived from fish bone mixed with chelated trace minerals and vitamins D3 (BCD) during 30 to 38 week-old.

**Items**	**BCD in diet (g/kg)**				**Pooled SEM**^1^	***P*-value**
	**0**	**0.5**	**1.0**	**2.0**		
Egg performance (*n* = 4)						
Hen-day egg production (%)	75.8	81.0	84.3	84.3	1.39	0.081
Average egg weight (g/egg)	67.8	66.3	66.5	66.8	0.26	0.193
Egg mass (g/b/d)	51.3	53.7	56.0	56.3	1.01	0.283
ADFI^2^(g/b/d)	137.5^b^	139.0^b^	141.0^b^	155.0^a^	2.56	0.036
FCR^3^	2.7	2.6	2.5	2.8	0.05	0.303
Egg quality (*n* = 64)						
Yolk color	12.7	12.3	12.6	12.3	0.11	0.397
Yolk ratio (%)	31.4	31.8	31.4	32.4	0.21	0.287
Albumen ratio (%)	56.6	56.0	56.5	55.3	0.23	0.152
Eggshell ratio (%)	12.2	12.2	12.1	12.3	0.07	0.798
Eggshell thickness (mm)	0.4	0.4	0.4	0.4	0.01	0.633

^1^Standard error of means.

^2^Average daily feed intake. ^3^Feed conversion ratio. ^a,b^Different superscripts in the same row express as significant value at *P* < 0.05.

The different BCD levels had no significant effects on egg quality parameters, such as yolk color, yolk ratio, albumen ratio, eggshell ratio, and eggshell thickness (Table 2). Similarly, the calcium and phosphorus contents of the eggshell and the tibia bone were not influenced (*P* > 0.05) by the dietary BCD (Table 3). The reason why no effect of dietary bio-calcium was found for the alteration of calcium and phosphorus contents in eggshell and tibia bone may be those birds able to regulate the level of calcium and phosphorus to meet the optimal requirements for the interaction of calcium crystals during bone and eggshell formation. Tibia bone weight and length did not differ (*P* > 0.05) among the 4 treatments. Our intention was to find a way to decrease the number of cracked eggs that cause economic losses for the egg industry. The current results show that the tibia bone (*P* = 0.006) and eggshell hardness (*P* = 0.025) significantly increased and correlated with increasing BCD levels (Table 3). Calcium is an essential element in bone and eggshell formation, and therefore, calcium deficiency in egg-type poultry is a general problem associated with lower bone mass and eggshell breakage. It has also been noted that complex organic calcium derived from grass fish bones can promote growth, serum calcium and alkaline phosphatase levels, and optimal femur weight in calcium-deficient rats (Tang et al., 2018). Jung & Kim (2007) noted that fish skeleton powder can be used as a bio-calcium, with high solubility and bioavailability. Similarly, piglets fed dietary salmon bones treated with enzymes as a source of bio-calcium showed greater calcium absorption than those fed diets supplemented with calcium carbonate (Malde et al., 2010). Due to the fact that BCD was produced from fish bone and primarily consists of bio-calcium with a highly bioavailability, it seems that the use of bio-calcium as a calcium source may be a novel choice to increase the amount of soluble calcium available for absorption via the intestinal mucosa. This hypothesis related with Benjakul et al. (2017) who described that bio-calcium derived from fish bone can be used as an alternative calcium dietary supplement due to a high calcium solubility in gastrointestinal tract, thereby an increasing amount of soluble calcium availability for absorption via the gut mucosa (Jung, Lee, & Kim, 2006).

However, calcium is not the only important nutrient for bone health and optimal eggshell characteristics; trace minerals, such as manganese, zinc, and copper, are also important. Majority of trace minerals are conventionally added in commercial poultry diet as the form of inorganic salts such as oxides, carbonates and sulfates. Trace minerals are essential for numerous biochemical functions and work within metabolic pathways as catalytic agents (Richards, 1997; Zhao et al., 2010) making them plays a critical role for optimal growth and normal development of bone morphology, feather structure, enzyme structure and function (Nollet, van der Klis, Lensing, & Spring, 2007). They have also proved to be crucial elements for bone and eggshell formation due to their influence on the formation of enzymes required during the mineralization process. Due to the nutritional antagonisms of trace mineral salts could occur in impaired absorption via the gastrointestinal tract (Aksu, Ozsoy, Aksu, Yoruk & Gul, 2011). However, chelated trace minerals have been accepted to use in the ration at a lower level as a high bioavailable mineral (Sun, Guo, Li, Zhang, & Wen, 2012; Ghasemi, Hajkhodadadi, Hafizi, Taherpour, & Nazaran, 2020). Upadhaya, Lee, Park & Kim (2016) noted that calcium digestibility and calcium level in blood increased in laying hens supplied drinking water supplemented with chelated water-soluble mineral such as zinc, manganese, and copper. Manangi, Vazques-anon, Richards, Carter, & Knight, 2015 reported that the eggs of 68-week-old laying hens increased in eggshell hardness after inorganic sulfates were completely replaced with chelated zinc-copper-magnesium in the laying diet. It appears that chelated trace minerals have greater bioavailability than inorganic trace minerals, resulting in increased eggshell strength. This was previously confirmed by Zhao et al. (2010), who published a study indicating that dietary chelated trace minerals (manganese, copper, and zinc) could benefit the footpad health of broilers. Vieira (2008) also described that chelated minerals and vitamin C are strongly associated with mineralized bone matrix. Laying hens provided diet containing manganese, zinc, and copper in the form of methionine hydroxy analog chelates showed an increased bioavailability and eggshell thickness compared with those fed the sulfates trace mineral (Sun, Guo, Li, Zhang, & Wen, 2012). Similarly, Chelated trace minerals could promote bone mineralization of broiler chickens (Ghasemi, Hajkhodadadi, Hafizi, Taherpour, & Nazaran, 2020). However, it is well known that a deficiency of these trace minerals (manganese, zinc, and copper) reduces the development and stability of collagen fibers, which negatively impacts the organic matrix structure, as well as the bone mineralization. This phenomenon results in skeletal abnormalities and eggshell deformities. In fact, although the dietary BCD did not influenced the calcium and phosphorus contents in eggshell and tibia bone, but the strongest tibia bone and eggshell were observed in the 2.0 g/kg BCD group when compared to the control group (*P* < 0.01). Because zinc and copper act as activator of collagen involved in bone development while manganese-dependent enzymes plays vital roles as the booster of the proteoglycan matrix formation in the cartilage model for developing bone (Richards, Zhao, Harrell, Atwell & Dibner, 2010). According to this results, it seems that chelated minerals might also have a beneficial effect on bone matrix and collagen fibers (Richards et al., 2010) as well as ossification and mineralization of the bone tissue (Kwiecień, Winiarska-Mieczan, Milczarek, Tomaszewska & Matras, 2016). Furthermore, these elements play a very important role in strength of bone and eggshell as enzymes activators such as lysyl oxidase, glycosyltransferase, and carbonic anhydrase (Akagawa, Wako & Suyama, 1999; Nys, Gautron, Garcia-ruiz & Hincke, 2004; Xiao et al., 2014) influencing the calcium crystals during bone and eggshell formation (Fernandes et al., 2008; Xiao et al., 2014).

**Fig. 1:**
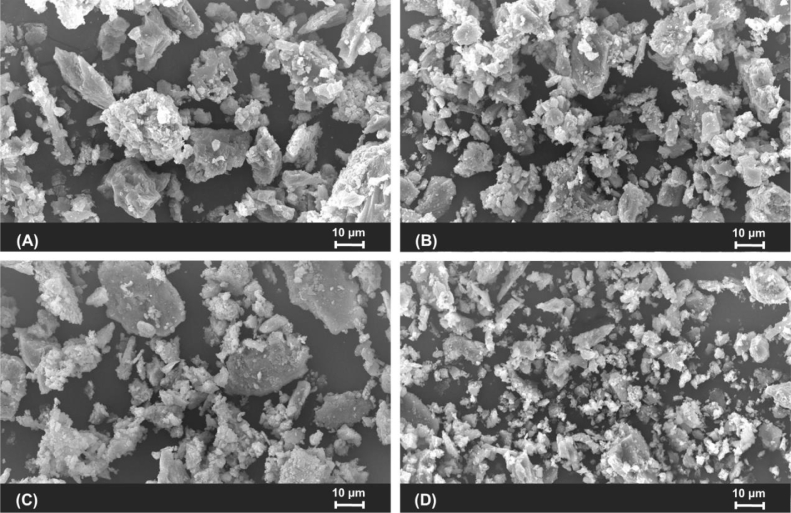
Scanning electron microscopic images of particle size distribution of ground eggshell samples in laying ducks fed a basal diet without supplement (A) and diets supplemented with bio-calcium derived from fish bone mixed with chelated trace minerals and vitamin D3 (BCD) at 0.5 (B), 1.0 (C), and 2.0 (D) g/kg during 30 to 38 week-old. Scale bar = 10 µm (1000 ×).

On the other hand, vitamin D3 has commonly been used in poultry production to encourage the proper metabolism of calcium and phosphorus and to maintain the skeletal system. Bouillon & Suda (2014) noted that vitamin D is considered an essential vitamin for calcium absorption and bone mineralization, as well as eggshell calcification. Also, vitamin D can contribute a vital role in bone metabolism (Rush et al., 2005), maintaining calcium and phosphorus homeostasis, skeletal health, and muscle development (Geng et al., 2018). Fig. 1 shows the SEM images of particle size distribution of ground eggshell samples in laying ducks fed a basal diet with and without BCD supplement. All samples (A–D) show a similar morphology and distribution, which implies that the particles were homogenous with non-uniform size. Ground eggshell samples of each group were dispersed on an aluminum stub with a rough surface. Results regarding the feeding of BCD in laying ducks show that it had no pronounced effect on the morphology of eggshell powder.

Because BCD mixture is a combination of bio-calcium, chelated trace minerals, and vitamin D3, it might have synergistic effects that increase the amount of soluble calcium available for absorption and enhance the bone mineralization and eggshell calcification. Thus, this study concludes that the inclusion of 2.0 g/kg BCD mixture in the laying duck diet can be a potential approach to improve tibia bone and eggshell hardness, without detrimental effect on egg performance. (Table 3)

**Table 3:** Hardness, calcium and phosphorus of eggshell and tibia bone in laying ducks fed dietary bio-calcium derived from fish bone mixed with chelated trace minerals and vitamins D3 (BCD) during 30 to 38 week-old.

**Items**	**BCD in diet (g/kg)**				**Pooled SEM**^1^	***P*-value**
	**0**	**0.5**	**1.0**	**2.0**		
Hardness (N)						
Eggshell	38.8^b^	41.6^ab^	41.2^ab^	43.9^a^	0.52	0.006
Tibia bone	119^b^	122^b^	140^ab^	155^a^	5.22	0.025
Calcium content (% DM)						
Eggshell	69.9	66.7	67.7	70.1	0.66	0.175
Tibia bone	41.6	39.2	39.3	35.5	0.96	0.154
Phosphorus content (% DM)						
Eggshell	0.4	0.5	0.5	0.5	0.04	0.747
Tibia bone	16.4	16.2	16.5	17.6	0.39	0.616
Tibia bone weight (g)	4.8	4.7	5.2	4.5	0.11	0.085
Tibia bone length (cm)	9.5	9.5	9.7	9.6	0.05	0.579

^a,b^Different superscripts in the same row express as significant value at *P* < 0.05.

^1^Standard error of means (*n*= 4 samples per treatment).

## Ethical approval

Ethical clearance was approved and regulated by the Naresuan University Agricultural Animal Care and Use Committee (NUAACUC; approval number: 62 01 003).

## Disclosure statement

No potential conflict of interest was reported by the authors.

## Declaration of Competing Interest

The authors declare the following financial interests/personal relationships which may be considered as potential competing interests: Tossaporn Incharoen reports financial support was provided by Agricultural Research Development Agency (ARDA).
